# Severe Peripheral Joint Laxity is a Distinctive Clinical Feature of Spondylodysplastic-Ehlers-Danlos Syndrome (EDS)-*B4GALT7* and Spondylodysplastic-EDS-*B3GALT6*

**DOI:** 10.3390/genes10100799

**Published:** 2019-10-12

**Authors:** Stefano Giuseppe Caraffi, Ilenia Maini, Ivan Ivanovski, Marzia Pollazzon, Sara Giangiobbe, Maurizia Valli, Antonio Rossi, Silvia Sassi, Silvia Faccioli, Maja Di Rocco, Cinzia Magnani, Belinda Campos-Xavier, Sheila Unger, Andrea Superti-Furga, Livia Garavelli

**Affiliations:** 1 Medical Genetics Unit, Maternal and Child Health Department, Azienda USL-IRCCS of Reggio Emilia, 42122 Reggio Emilia, Italy; stefanogiuseppe.caraffi@ausl.re.it (S.G.C.); ivan.ivanovski@ausl.re.it (I.I.);; 2 Child Neuropsychiatry Unit, Azienda USL of Parma, 43125 Parma, Italy; 3 Department of Surgical, Medical, Dental and Morphological Sciences with interest in Transplant, Oncology and Regenerative Medicine, University of Modena and Reggio Emilia, 42121 Reggio Emilia, Italy; 4 Department of Molecular Medicine, Unit of Biochemistry, University of Pavia, 27100 Pavia, Italy; 5 Rehabilitation Pediatric Unit, Azienda USL-IRCCS of Reggio Emilia, Reggio Emilia, 42122 Reggio Emilia, Italy; 6 Department of Pediatrics, Unit of Rare Diseases, IRCCS Istituto Giannina Gaslini, 16147 Genoa, Italy; 7 Neonatology and Neonatal Intensive Care Unit, Maternal and Child Department, University of Parma, 43121 Parma, Italy; 8 Division of Genetic Medicine, Centre Hospitalier Universitaire Vaudois (CHUV), University of Lausanne, 1011 Lausanne, Switzerland

**Keywords:** beta-1,3-galactosyltransferase 6 (*B3GALT6)*, beta-1,4-galactosyltransferase 7 (*B4GALT7)*, spondyloepimetaphyseal dysplasia with joint laxity (SEMDJL1, SEMDJL-Beighton type), Ehlers–Danlos syndrome (EDS), spondylodysplastic Ehlers–Danlos syndrome (spEDS), spEDS-*B3GALT6*, spEDS-*B4GALT7*, extreme laxity of distal joints, soft, doughy skin on the hands and feet

## Abstract

Variations in genes encoding for the enzymes responsible for synthesizing the linker region of proteoglycans may result in recessive conditions known as “linkeropathies”. The two phenotypes related to mutations in genes *B4GALT7* and *B3GALT6* (encoding for galactosyltransferase I and II respectively) are similar, characterized by short stature, hypotonia, joint hypermobility, skeletal features and a suggestive face with prominent forehead, thin soft tissue and prominent eyes. The most outstanding feature of these disorders is the combination of severe connective tissue involvement, often manifesting in newborns and infants, and skeletal dysplasia that becomes apparent during childhood. Here, we intend to more accurately define some of the clinical features of *B4GALT7* and *B3GALT6*-related conditions and underline the extreme hypermobility of distal joints and the soft, doughy skin on the hands and feet as features that may be useful as the first clues for a correct diagnosis.

## 1. Introduction

The extracellular matrix (ECM) of connective tissues like cartilage, bone, tendon, ligaments and skin is a complex interacting arrangement of proteins, glycoproteins and proteoglycans (PGs) that forms the scaffold of the human body and also constitutes the vital environment supporting cell growth and differentiation. PGs are macromolecules that are complex in their structures and represent an important component of both the ECM and of cell membranes. PGs have mechanical and osmotic functions but are strongly involved in signaling pathways controlling cell-to-cell interactions as well as cell growth and differentiation and tissue repair [1,2,3]. PGs are composed of a core protein and one or more glycosaminoglycan (GAG) chains that are long polysaccharides consisting of repeating disaccharide units. On the basis of the composition of these units, the PGs are subdivided into heparan sulfate (HS) PGs and chondroitin sulfate (CS)/dermatan sulfate (DS) PGs [4]. The biosynthesis of the GAG side chain begins with the creation of a common tetrasaccharide, a so called “linker”, covalently linked to the hydroxyl group of serine residues on the core protein (O-glycosylation).

**Figure 1:**
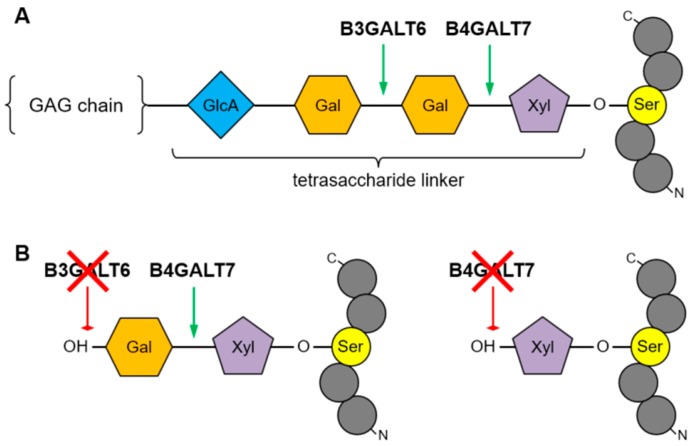
**A**. Galactosyltransferases I (B4GALT7) and II (B3GALT6) catalyze the addition of galactose units at specific positions of the tetrasaccharide region linking Serine residues on the protein backbone to glycosaminoglycan (GAG) chains. Xyl = Xylose, Gal = Galactose, GlcA = Glucuronic Acid. **B**. Functional defects of B4GALT6 or B4GALT7 inhibit addition of one or both Gal monosaccharides, respectively, and prevent GAG chain polymerization.

The linker region is synthesized through a coordinated mechanism involving specific glycosyltransferases. It starts with the transfer of a xylose residue by xylosyltransferase I or II (encoded by *XYLT1*, OMIM *608124, and *XYLT2*, OMIM *608125), followed by the addition of two galactose residues by galactosyltransferase I, encoded by *B4GALT7* (OMIM 604327) and galactosyltransferase II encoded by *B3GALT6* (OMIM *615291) (Figure 1). Subsequently there will be a transfer of glucuronic acid by glucuronosyltransferase I encoded by *B3GAT3* (OMIM *606374). The so-called “linkeropathies” are recessive conditions caused by variations in genes encoding the enzymes responsible for the synthesis of the linker region of PGs. 

Variants of the *XYLT1* gene are responsible for skeletal dysplasias, including Baratela–Scott syndrome (OMIM 615777, formerly erroneously called Desbuquois dysplasia type 2) [5,6,7,8,9,10], variants of *XYLT2* are the cause of the spondylo-ocular syndrome (OMIM 605822) [11,12,13], while variants of *B3GAT3* cause a Larsen-like syndrome and a more severe skeletal dysplasia (OMIM 245600) [14,15,16,17,18,19,20]. 

In particular, variants in the *B4GALT7* and *B3GALT6* genes are the cause of a combined skeletal and connective tissue phenotype.

A *B4GALT7*-related condition was initially described as “progeroid type 1” Ehlers–Danlos syndrome (EDS), due to the phenotype characterized by premature aging and loose elastic skin in one reported patient [21,22,23,24,25,26,27,28,29,30]. Subsequently, Cartault et al. in 2015 described a unique homozygous *B4GALT7* mutation, p.Arg270Cys, in a large cohort of patients affected by the so-called Larsen of Reunion Island syndrome [31]. This contributed to define the currently known phenotype of *B4GALT7*-related EDS, with round flat face, proptosis, short stature, hypotonia, radioulnar synostosis, osteopenia, hyperextensible skin and joint hypermobility (OMIM #130070) [24,25,31,32,33].

*B3GALT6* variants cause a single disorder that was alternatively described as spondyloepimetaphyseal dysplasia (SEMD) with joint hypermobility (SEMDJL1 or SEMDJL Beighton type; OMIM #271640) in the “Nosology and classification of genetic skeletal disorders: 2015 revision” [34,35,36,37,38,39,40,41,42,43] or as severe EDS-like disorder (OMIM #615349) because of the striking joint laxity and muscular hypotonia in infancy [44,45]. Affected patients generally present clinical features of connective tissue weakness in infancy, and subsequently develop signs of skeletal dysplasia; sometimes these signs are already present at birth. Some patients develop life-threatening complications, such as aortic dilatation, aneurysms and cervical spine instability.

Due to the mixed phenotype, with signs of EDS and signs of skeletal dysplasia, the *B4GALT7* and *B3GALT6*-related syndromes have been called ”spondylodysplastic EDS” (spEDS) in the recently revised EDS classification of 2017 [46,47,48].

Hereby we present the clinical features of 3 patients with spEDS. Two are novel patients: Pt. 1 with *B4GALT7*-related EDS, and Pt. 3 with a severe *B3GALT6*-related EDS. Pt. 2 was originally reported as *B3GALT6*-related SEMDJL by Nakajima et al. in 2013, but is included here because his clinical description was largely unpublished and his condition has undergone a significant evolution. Through the description of these patients and a review of previous literature reports, we contribute to a more accurate definition of the clinical features associated with *B4GALT7*- and *B3GALT6*-related syndromes. In particular, we point out the extreme distal joint hypermobility and skin hyperextensibility (more evident in hands and feet) these two conditions have in common, and the peculiar radiological signs of each syndrome. The combination of these features is the main indicator for clinicians to suspect these ultra-rare conditions.

## 2. Materials and Methods

### 2.1. Metabolic Labeling of Fibroblast Cultures and PG Synthesis Analysis

Skin fibroblasts from the patient and controls were cultured in minimum essential medium (MEM) with 10% fetal calf serum and antibiotics at 37 °C in a humidified atmosphere containing 5% CO_2_. Confluent cells in 10-cm^2^ petri dishes were preincubated for 4 h with or without 1 mM p-nitrophenyl *β*-D-xylopyranoside in MEM containing 250 *μ*M cold Na_2_SO_4_ without FCS in 5% CO_2_ at 37°C. Cells were then labeled with 150 *μ*Ci/ml Na_2_[^35^S]O_4_ in the same medium for 24 h, as described previously [49]. At the end of the labeling period, an equal volume of 100 mM sodium acetate buffer, pH 5.8, containing 8 M urea, 4% Triton X-100, 20 mM ethylenediaminetetraacetic acid, 20 mM N-ethylmaleimide, and 1mM phenylmethanesulfonyl fluoride was added to the medium. The cell layer was harvested in 50 mM sodium acetate buffer, pH 5.8, containing 2 M urea, 2% Triton X-100, and an aliquot was used for protein content determination with the bicinchoninic acid Protein Assay (Pierce), while the remainder was added to the medium. Samples were loaded on 1 ml DEAE Sephacel^TM^ columns; after column washing with 50 mM sodium acetate buffer, pH 6.0, 8 M urea, 0.15 M NaCl, 0.5% Triton X-100 and proteinase inhibitors, PGs were eluted with 1 M NaCl in the same buffer, recovered by precipitation with 9 volumes of ethanol. The pellet was washed with 70% ethanol and then solubilized in water; PGs were quantified by measuring the ^35^S-activity using a liquid scintillation counter and normalized to the protein content.

Labeled PGs synthesized by cells in absence of p-nitrophenyl *β*-D-xylopiranoside and purified as described above, were lyophilized, dissolved in 4 M guanidinium chloride, 50 mM sodium acetate buffer, pH 6.0, 0.5% Triton X-100, and analyzed by Size Exclusion Chromatography. Samples were then loaded on a Superose 6 10/300GL column (GE Healthcare) and eluted in the same buffer at 0.2 ml/min. Fractions of 0.4 ml were collected and ^35^S-activity was measured by scintillation counting [50].

### 2.2. Molecular Genetic Testing

Genomic DNA of each patient was isolated from peripheral blood mononuclear cells and analyzed using a capture-based IonAmpliSeq custom panel on an Ion Torrent series S5 instrument (Thermo Fisher Scientific). Results were filtered for the *B4GALT7* and *B3GALT6* genes. Identified variants were confirmed by PCR amplification and subsequent bidirectional Sanger sequencing, using primers encompassing the entire exon and intron–exon boundaries comprising the variants in the case of *B4GALT7*, or the entire coding sequence and surrounding untranslated regions in the case of *B3GALT6* single exon. In parallel, DNA samples from the respective parents were analyzed by Sanger sequencing in order to evaluate parental segregation. Primer sequences are available upon request.

## 3. Patients and Results

### 3.1. First Patient

#### 3.1.1. Clinical Report

**Figure 2:**
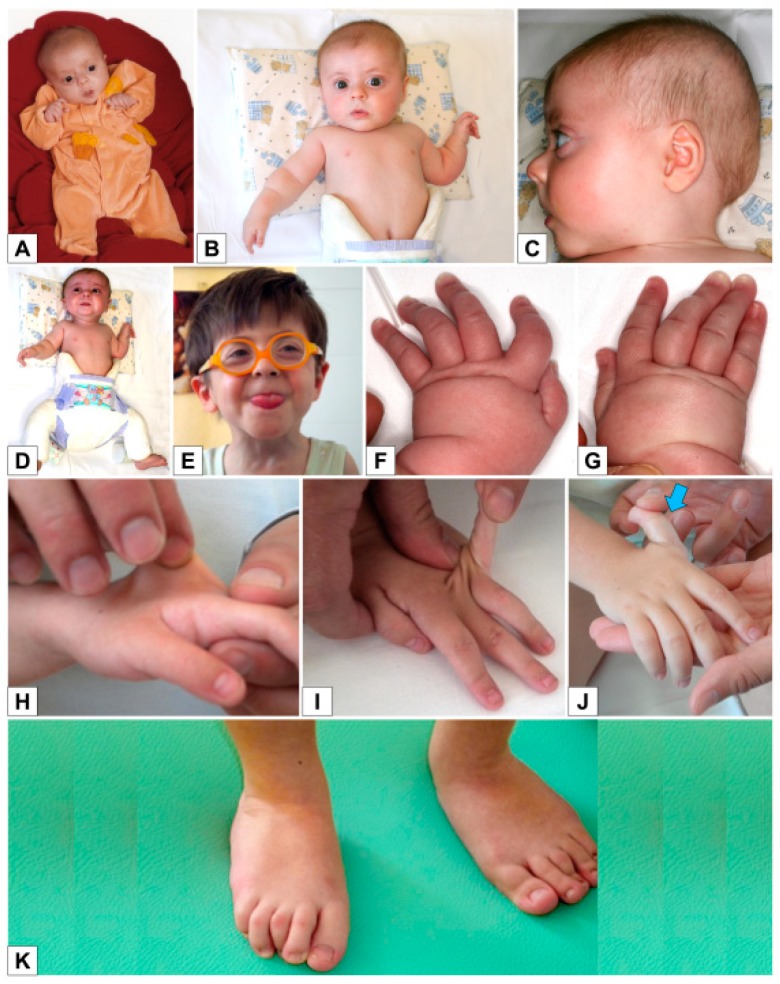
Patient 1 **A**) Age 3 months: ulnar deviation of fingers; **B**) Age 3 months: round flat face, mild proptosis, mesomelic shortening of upper limbs, folded skin on forearm; **C**) Age 3 months: flat profile; **D, F–G**) Age 3 months: ulnar deviation of fingers, 2nd finger clinodactyly, 2nd–5th finger camptodactyly on the left hand, 3rd and 4th finger camptodactyly on the right hand; **E**) Age 3 years 7 months: round face, blue sclerae, proptosis; **H**–**K**) Age 3 years 7 months: soft, hyperextensible skin and extreme joint hypermobility (Beighton score: 5) in particular of hands and feet; significant improvement in finger ulnar deviation, clinodactyly, camptodactyly of the second finger, and overlapping toes.

The boy is the first child of healthy, non-consanguineous parents. The pregnancy was unremarkable and the mother was not exposed to any known teratogens during pregnancy. Fetal movements were described as normal. He was born by cesarean delivery at week 38 with birth weight of 2520 g (3rd–10th centile), length 43 cm (<3rd centile), and head circumference of 32 cm (3rd–10th centile). Apgar scores were 9/10. He presented with mesomelic shortening of upper limbs, ulnar deviation of fingers and left hip dysplasia. Cerebral and renal ultrasound were normal, while echocardiography showed atrial septal defect. The baby was discharged from hospital at the age of 6 days, with diagnosis of “arthrogryposis with ulnar deviation of fingers, left hip dysplasia, and congenital heart disease”, and later he was fitted with a finger extension device and was given pelvic bimalleolar hip plaster for left hip dysplasia. He was seen at our Medical Genetics Unit at 3 months of age (Figure 2): length and weight, calculated by taking into account the hip plaster, were respectively 51 cm (3rd–10th centile) and 4900 g (3rd centile), and head circumference was 41 cm (25th centile). The physical examination showed: round flat face, mild proptosis, slightly blue sclerae (Figure 2A–D), joint hypermobility especially evident in the hands, mesomelic shortening of the arms, abnormal prono-supination of the left upper limbs, often flexed, short hands and feet with soft skin, bilateral ulnar deviation of fingers, 2nd finger clinodactyly, 2nd–5th finger camptodactyly on the left, and 3rd and 4th finger camptodactyly on the right (Figure 2F–G). 

During the clinical follow-up, we noticed an improvement in the finger alterations, but a worsening of the cutaneous hyperextensibility and joint hypermobility. At the age of 3 years and 7 months, he presented with round face, blue sclerae, proptosis (Figure 2D), soft hyperextensible skin and extreme joint hypermobility (Beighton score: 5) in particular of hands and feet, with significant improvement in finger ulnar deviation, clinodactyly, camptodactyly and overlapping toes (Figure 2H–K). Height and weight were 87 cm (<3rd centile) and 9920 g (<3rd centile), head circumference was 49 cm (10th centile). Span was 82 cm and span/height ratio was 0.94. Early psychomotor development was delayed at least in part owing to his orthopedic conditions. He gained head control at 3 months, was able to sit at 3 years and walk aided at the age of 4 years. He uttered his first words at 2 years, but his language skills improved rapidly and significantly thereafter: at 5 years, psychological evaluation reported an IQ of 105.

**Figure 3:**
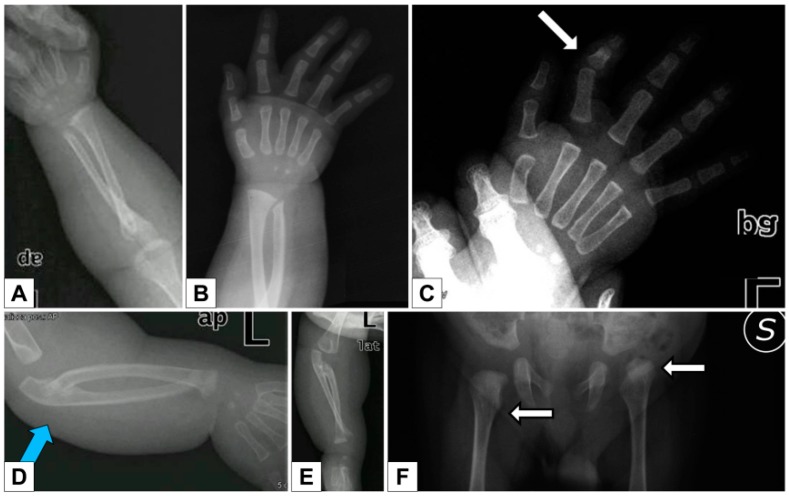
Patient 1 **A**–**B**, **D**–**E**) Bilateral bowing of ulna and radius with dislocation/subluxation and radioulnar synostosis, metaphyseal widening of the radius; **C**) bilateral short and dysmorphic 2nd finger middle phalanx, short first metacarpal; **F**) hip dysplasia.

The skeletal X-rays demonstrated wide anterior ribs, bilateral bowed ulna and radius with dislocation/subluxation and radioulnar synostosis, bilateral metaphyseal widening of the radius, bilateral short and dysmorphic 2nd finger middle phalanx, short first metacarpal (Figure 3A–F). 

Electrencephalography, electromyography and brain magnetic resonance imaging were all normal. At the age of 5 years, molecular analysis of *B4GALT7* was performed, showing the presence of two novel pathogenic mutations, indicative of spEDS.

#### 3.1.2. Molecular and Functional Analysis

**Figure 4:**
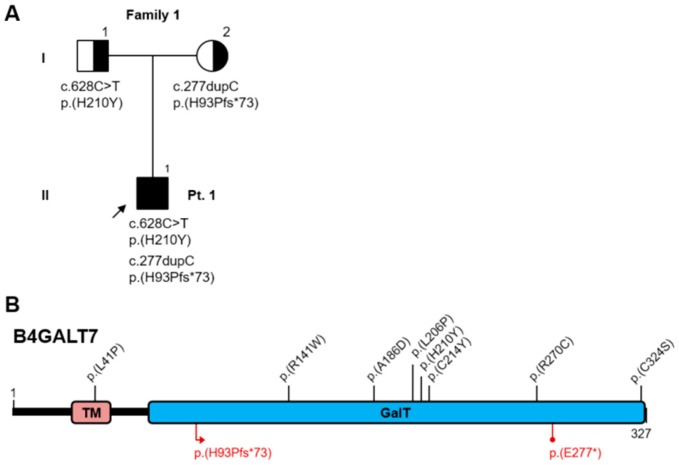
**A**) Pedigree of Family 1; **B**) Schematic representation of the B4GALT7 protein: TM = transmembrane domain, GalT = Galactosyl-transferase catalytic domain. Protein variants reported in the literature are annotated in the cartoon (see Table 1 for references). Top half: missense variants (black). Bottom half: truncating variants (red).

Karyotype was normal, 46,XY. Our first diagnostic hypothesis was either myopathy or collagen VI pathology, but molecular analysis of the *COL6A1*, *COL6A2*, *COLA613*, *RAPSN* genes did not reveal any rare or pathogenic variant. Subsequently, by analyzing a panel of EDS-related genes, two variants were identified in compound heterozygosity in exons 2 and 3 of the *B4GALT7* gene: NM_007255:c.[277dupC];[628C>T], NP_009186:p.[(His93Profs*73)];[(His210Tyr)] (Figure 4).

The single nucleotide insertion c.277dupC, inherited from the mother, creates a new reading frame, terminating with a premature stop codon 73 residues downstream, and its transcript is predicted to undergo nonsense-mediated decay (https://nmdprediction.shinyapps.io/nmdescpredictor/). This variant was also described in another patient by Salter et al. in 2016 [32]. The heterozygous C>T transition at nucleotide 628, inherited from the father, results in a non-conservative Histidine to Tyrosine substitution at codon 210 and occurs in a highly conserved residue. This variant has not been reported before, but it is considered deleterious by multiple prediction algorithms (MutationTaster, PolyPhen2, SIFT) and is absent from reference population databases (gnomAD, 1000 Genomes). Based on these criteria and on the clinical features of the patient, we assume that the combination of these variants in trans is causative.

The study of PG synthesis on patient’s fibroblasts obtained by skin biopsy did not reveal any significant differences compared to normal controls, neither at basal conditions nor in the presence of beta-xyloside to enhance glycosaminoglycan synthesis. However, the level of PG synthesis in the patient’s fibroblasts was at the lower normal limit. Analysis through gel filtration to assess the fraction of whole PGs with respect to glycosaminoglycans did not show any differences. Although these results were not significant *per se*, the slightly reduced PG levels combined with the genetic testing offered some indication that the variants identified in *B4GALT7* may in fact have a deleterious effect on protein function. 

### 3.2. Second Patient

#### 3.2.1. Clinical Report

This patient was partially described in Nakajima et al., 2013 as P9 [45]. He is the first child of healthy non-consanguineous parents, born at the 40th week of gestation by vaginal delivery. During pregnancy, no known exposures to potential teratogens were detected. The mother reported normal fetal movements. Prenatal ultrasound showed bilateral megaureter. At birth, weight was 2870 g (10th centile), length was 48 cm (3rd–10th centile) and head circumference measured 34 cm (25th centile). He presented with: joint hypermobility, soft skin, bilateral congenital talipes equinovarus and right hip dysplasia, which was treated with a Pavlik harness. Renal ultrasound showed a mild bilateral caliceal dilatation, while renal scintigraphy was normal. During the first year of life he was diagnosed with early-onset scoliosis, and he began to wear an orthopedic corset from the age of 4 years. Two years later. he was operated for scoliosis after correction with Halo Gravity Traction and he subsequently received three operations for the lengthening of the pins (until the age of 10 years). He suffered from frequent fractures: a fracture of the left femur after a low trauma at the age of 5 years and 6 months and to the right femur at the age of 8 years and 8 months and a micro-fracture of the right proximal fibula in an area of reduced bone density, compatible with an osseous cyst, at the age of 10 years. Furthermore, he had recurrent luxation of the toes. 

Early psychomotor development was normal, but he later suffered from motor delay due to his skeletal and large joint anomalies. He gained head control at the age of 2 months and could sit up at the age of 8 months. He could walk unaided at the age of 2 years and uttered his first words at the age of 1 year. He currently attends the first year of middle school with a support teacher owing to a diminished ability to concentrate. He does not present with intellectual disability or other behavioral disturbances.

**Figure 5:**
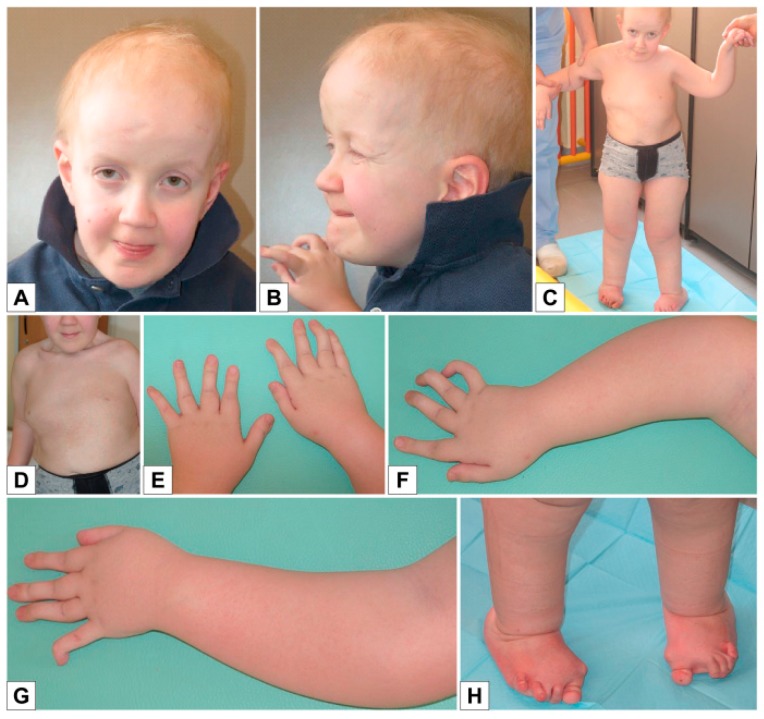
Patient 2 **A**–**C**) Sparse hair, high and prominent forehead, sparse eyebrows, deeply-set eyes, blue sclerae, hypoplastic columella, large ears; **D**–**H**) Thin, pale, extremely soft skin, with prominent veins on the trunk and limbs, limited elbow extension, skin hyperextensibility and distal joint hypermobility especially of the hands, long and tapered fingers. Feet: hypoplastic nails, short, overlapping toes, hallux valgus.

At the age of 12 years and 7 months (Figure 5), height was 109cm (<3rd centile <-6SD), weight 29 kg (<3rd centile), head circumference 54 cm (50th–75th centile), span 114 cm, span/height ratio 1.06. Pubertal stage was A0P1B1, with testicular volume 1–2 ml on the left side and 2–3ml on the right. He had sparse hair, high and prominent forehead, large ears, sparse eyebrows, large deeply-set eyes, blue-gray sclerae, hypoplastic columella and misalignment of teeth, which were small, with abnormal enamel and yellow-brown discoloration (Figure 5A–C). He had thin, pale, extremely soft skin, with a prominent venous patterning on the trunk and limbs, limited elbow extension, skin hyperextensibility and distal joint hypermobility especially of the hands (Beighton scale: 6), long and tapered fingers, normal nails. His feet had hypoplastic nails, short and overlapping toes, hallux valgus (Figure 5D–H).

**Figure 6:**
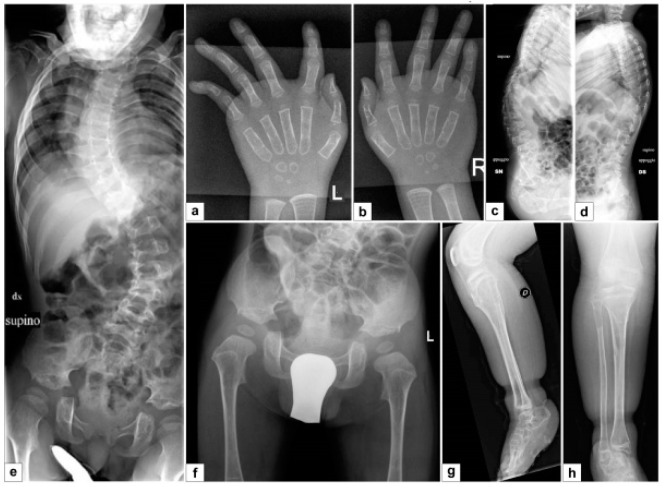
Patient 2 **A**–**H**) Severe, early onset kyphoscoliosis, osteopenia, thin metacarpals and phalanges.

The skeletal X-rays demonstrated severe kyphoscoliosis, osteopenia, thin metacarpals and phalanges (Figure 6A–H). 

Audiometric and ophthalmological evaluations were normal. Abdominal ultrasound showed ptosic kidneys, bilateral pelvic ureteral dilatation and thickening of the bladder walls. Echocardiography demonstrated mild dilatation of the aortic bulb and mild mitral valve prolapse.

#### 3.2.2. Molecular Analysis

**Figure 7:**
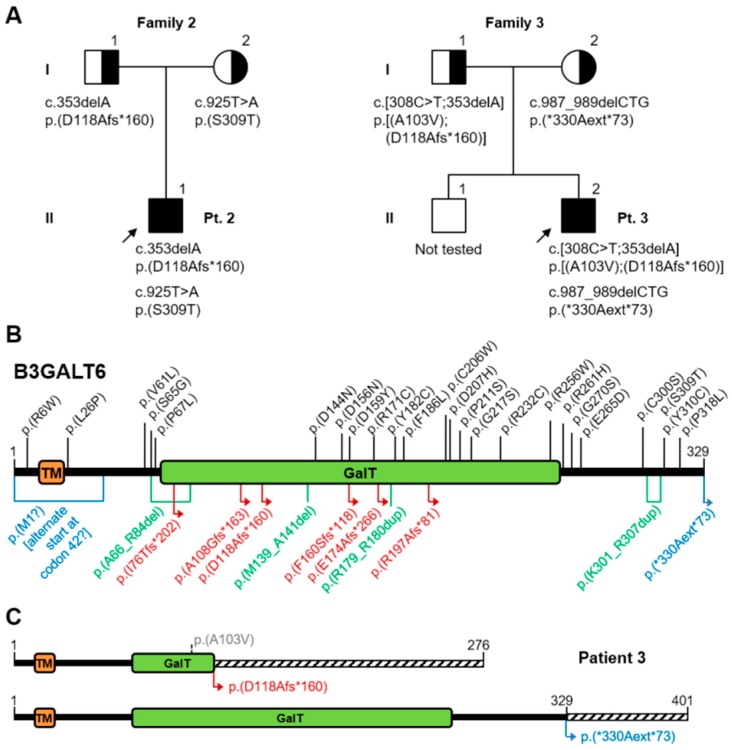
**A**) Pedigree of Families 2 and 3; **B**) Schematic representation of the B3GALT6 protein: TM = transmembrane domain, GalT = Galactosyl-transferase catalytic domain. Protein variants reported in the literature are annotated in the cartoon (see Table 2 for references). Top half: missense variants (black). Bottom half: frameshift variants (red), in-frame indels (green), other length-altering variants (blue); **C**) Protein projection of the two alleles from Pt.3; dashed boxes indicate an altered or extended reading frame.

Sanger sequencing confirmed the two previously reported variants in the single exon of the *B3GALT6* gene [45]: NM_080605:c.[353delA];[925T>A], NP_542172:p.[(Asp118Alafs*160)];[(Ser309Thr)] (Figure 7A,B). 

The frameshift variant was inherited from the father and the missense variant from the mother. Both variants can be considered pathogenic, according to the predicted effect on the protein, the consistency of the clinical phenotypes, and their rarity in reference population databases such as gnomAD (MAF=0.0026% and absent, respectively). Variant c.353delA has also been found in our third patient (reported below), while variant c.925T>A had already been reported in other patients in the literature [45,48]. Since this gene is encoded by a single exon, its frameshift variants, identified in various patients, are expected to escape nonsense-mediated mRNA decay. They probably exert a pathogenic effect by leading to an unstable transcript or an unstable/non-functional protein, as suggested by experimental evidence [48].

### 3.3. Third Patient

#### 3.3.1. Clinical Report

He is the second child of non-consanguineous parents, born at 35th week of gestation by caesarean section, owing to the premature rupture of the amniotic sac. During pregnancy, no known exposures to potential teratogens were detected. Prenatal ultrasound indicated bilateral talipes equinovarus. The mother reported normal fetal movements. At birth, weight was 2500 g (50th centile), length was 47 cm (50th–75th centile) and head circumference measured 34 cm (90th centile). Apgar scores were 1’:7 5’:9. He presented with joint hypermobility, soft skin, adducted thumbs, right talipes equinovarus. In the second year of life he was diagnosed with early-onset scoliosis, and he began to wear an orthopedic corset. 

At age 6 years, he was operated for bilateral cryptorchidism and at age 9 years, he underwent a gastrostomy to correct Barrett’s oesophagus.

His motor development was delayed. He gained head control at the age of 4–5 months and could sit up at the age of 24 months. He could walk aided at the age of 5 years, he was able to walk alone at the age of 6 years and 6 months and uttered his first words at the age of 1 year. He currently attends the second year of middle school with a support teacher owing to a diminished ability to concentrate. He does not present with intellectual disability or other behavioral disturbances.

**Figure 8:**
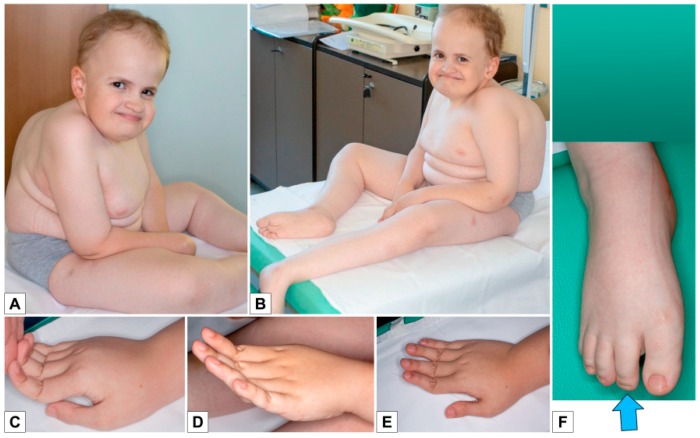
Patient 3 **A**,**B**) Sparse hair, high and prominent forehead with high hairline, mild bi-temporal depression, fairly large ears, blue-gray sclerae, malar hypoplasia, hypoplastic columella, short philtrum, thin, pale, soft skin with prominent veins on the trunk; **C**–**E**) Skin hyperextensibility and distal joint hypermobility especially of the hands, long and tapered fingers, with a tendency to ulnar deviation; **F**) Feet: hypoplastic nails.

Examination at the age of 12 years and 11 months showed (Figure 8A–F): height 123 cm (<<3rd centile), weight 28 kg (<3rd centile), head circumference 52 cm (10th centile), span 113 cm, span/height ratio 0.91. Pubertal stage were A0P1B1, with testicular volume of 1-2 ml bilaterally. He had sparse hair, high and prominent forehead with high hairline, mild bitemporal depression, fairly large ears, blue-gray sclerae, malar hypoplasia, hypoplastic columella, short philtrum, small misaligned teeth in the lower jaw, flat palate with longitudinal median mucous thickening (Figure 8A–B). He had thin, pale, soft skin, with slightly prominent veins on trunk, limited elbow extension, skin hyperextensibility and distal joint hypermobility especially of the hands (Beighton scale: 6), long and tapered fingers with a tendency to ulnar deviation (Figure 8C–E), hypoplastic nails on the feet (Figure 8F).

**Figure 9:**
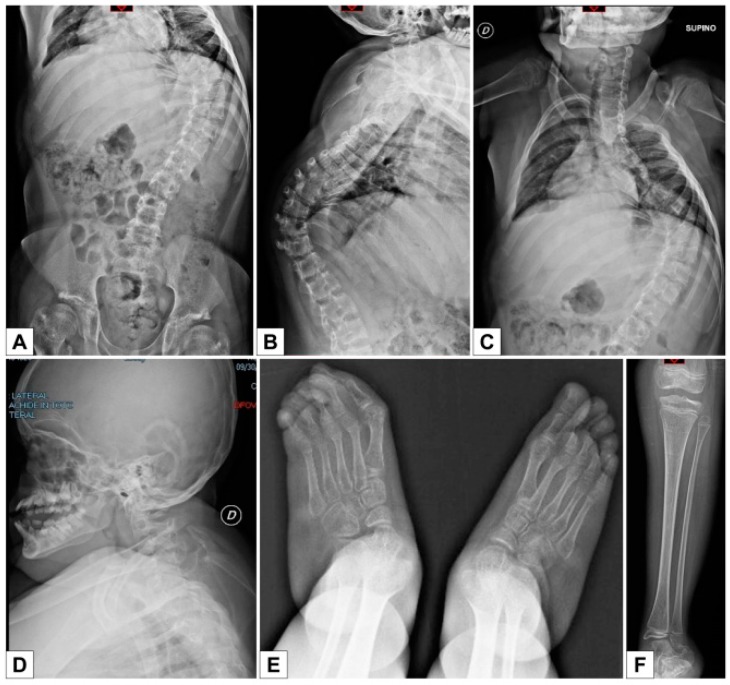
Patient 3 **A**–**F**) Severe early onset kyphoscoliosis; osteopenia of the acetabula, femur, tibiae and fibulae; thin metacarpals, metatarsals and phalanges.

Skeletal X-rays demonstrated severe kyphoscoliosis, osteopenia (especially in the acetabula, femurs, tibiae and fibulae), and thin metacarpals, metatarsals and phalanges (Figure 9A–F). 

Audiometric and ophthalmological evaluations were normal. Abdominal ultrasound was normal. Echocardiography demonstrated an atrial septal defect and mild mitralic insufficiency with thickened mitralic valve.

#### 3.3.2. Molecular Analysis

Karyotype was normal, 46XY. Genetic testing revealed three variants in the *B3GALT6* gene: NM_080605:c.[308C>T;353delA];[987_989delCTG], NP_542172:p.[(Ala103Val);(Asp118Alafs*160)];[(*330Alaext*73)] (Figure 7A–C).

Variant c.353delA, already described as pathogenic (patient 2 and Nakajima et al., 2013 [45]), has been found in *cis* with variant c.308C>T, both inherited from the healthy father. This missense variant is absent from the reference population databases (gnomAD, 1000 genomes), but occurs at a poorly conserved position and is predicted as benign by multiple algorithms (MutationTaster, PolyPhen2, SIFT). It should be classified as a variant of uncertain significance, as there is not enough evidence to suggest whether this variant alone would be tolerated or damaging to protein function, or to indicate a possible contribution to the deleterious effect of the frameshift variant.

Variant c.987_989delCTG, inherited from the healthy mother, disrupts the constitutive stop codon leading to an extended open reading frame, encoding a 72aa longer protein. It is absent from reference population databases (gnomAD, 1000 genomes), and by analogy with the frameshift variants in this gene, it is expected to produce an unstable or non-functional protein. Since it was found in trans with a recognized deleterious mutation, this variant can be considered as likely pathogenic.

## 4. Discussion

Biallelic variants in the *B4GALT7* and *B3GALT6* genes are responsible for conditions characterized by a combined skeletal and connective tissue phenotype. Their recent classification as spEDS [46] provides a valid assistance for their diagnosis, but the observation of new patients and the study of the clinical signs of those already reported in the literature can further improve our knowledge. In an effort to better define both the similarities and the peculiarities of these syndromes, we reviewed the notable features of our three patients and compared them to those of molecularly confirmed *B4GALT7*- and *B3GALT6*-related cases reported in the literature (Table 1 and Table 2) [18,21,22,23,24,29,30,31,32,33,44,45,48,51,52,53,54,55,56]

All three of our patients display a remarkably short stature. Pt. 2 and 3 are more severely affected, in part because of their kyphoscoliosis, with a stature progressing from moderately short (or normal for Pt. 3) at birth to well below the 3^rd^ percentile during childhood. Nearly all patients reported in the literature (65/66 evaluated cases) share this feature, confirming short stature as a main aspect of spEDS.

Joint hypermobility and soft, doughy and hyperextensible skin are some of the most striking characteristics in our patients. These qualities are particularly remarkable in the hands and, to a lesser extent, in the feet, and helped us restrict the diagnosis to an EDS-related condition. Since these features are shared by more than 90% of cases reported in the literature (63/66 and 62/68, respectively), in our opinion their combination should be considered a major diagnostic criterion for spEDS.

Craniofacial features for both *B4GALT7*- and *B3GALT6*-related conditions are quite variable among our patients and the reported individuals for whom either pictures or descriptive data could be evaluated, and do not seem suggestive of a facial gestalt. There are, however, some recurring features that may sometimes help to set the two conditions apart: i. proptosis is more frequent in spEDS-*B4GALT7* (32/33 vs 20/32); ii. midface retrusion has been frequently noted in both conditions, but in spEDS-*B3GALT6* it is often associated with a prominent forehead (31/38), sometimes emphasized by sparse hair (12/26) as in the case of our Pt. 2 and 3, while it would be more appropriate to state that spEDS-*B4GALT7* patients have an entirely flat face, usually short or round (31/33) and with a narrow mouth (31/33). The progeroid aspect of spEDS-*B4GALT7* described in Kresse’s child [24] and previously pointed out by Hernandez [21,22,23] was absent in all other patients, suggesting that it is not strongly associated with mutations in *B4GALT7*. As current terminology indicates, a progeroid facial appearance is the subjective interpretation of a series of facial features which should rather be evaluated individually. Some features such as thin skin or excessive wrinkles may occasionally appear in the description of the cases we reviewed, but never in a combination suggestive of premature aging, and none of these were observed in our patient. Therefore, as already suggested by other authors, we endorse the necessity to remove the term “progeroid type” from this syndrome, because it could be misleading.

The radiological findings are undoubtedly the most significant evidence in distinguishing *B4GALT7*-related and *B3GALT6*-related spEDS: the latter includes more numerous and pronounced elements of skeletal dysplasia, and for this reason has often been classified as such in the past. Radioulnar synostosis has been observed recurrently and almost exclusively in spEDS-*B4GALT7* (19/32 cases, including our patient, vs 3 documented cases of spEDS-*B3GALT6* [53,56]. On the other hand, most of the individuals affected by *B3GALT6*-related spEDS, including our patients, display severe kyphoscoliosis, usually congenital or early onset and progressive (36/39), and several of the skeletal changes associated with SEMDJL1, such as platyspondyly, short iliac bones, elbow dislocation with misalignment of the long bones (33/36). Osteopenia has been noted in both conditions. It has been reported more frequently in *B3GALT6*-related cases (22/26), usually in conjunction with fractures and luxations, as in the case of our patients. In *B4GALT7*-related cases there are significantly fewer records of this feature, but in most reports its absence or presence cannot be verified. Therefore, the actual prevalence of osteopenia is unknown and should probably deserve more consideration when referring spEDS suspects for a radiological exam.

A consequence of skeletal involvement (bowing of limbs) is considered one of the main criteria for suspecting spEDS, and it is possibly more common in the spEDS-*B4GALT7* series (30/32 vs 16/20); in fact, our Pt. 2 and 3 do not have this phenotype. Joint contractures of the upper limbs have been noted for both conditions, though they are usually reported at the elbow in spEDS-*B4GALT7* and at the hands in spEDS-*B3GALT6*. In our experience, it was actually our *B3GALT6*-mutated patients who displayed limited elbow extension, but this is a non-specific clinical sign that is often found in skeletal dysplasia.

Other clinical features appear to be peculiar of *B3GALT6*-related spEDS. The most notable are talipes equinovarus (23/37 patients, including ours), possibly with hypoplastic nails (cfr EDS classification of 2017 [46] and our patients 2 and 3), and abnormalities of the dentition (13/17 patients, including ours), sometimes with yellow-brown discoloration of the teeth (also observed in our Pt. 2).

Motor delay has been noted in the majority of reports in which developmental milestones were evaluated (27/36), including our three patients. It is most likely related to the recurrent skeletal anomalies (bowing of limbs, scoliosis, hip dysplasia) as well as to the frequent reports of hypotonia, also observed in all of our patients (36/48 evaluated cases). In order to overcome the difficulties in sitting and walking, these children often need assistance in the form of rehabilitation with appropriate physiotherapeutic interventions. Depending on the severity of the musculoskeletal features, some individuals may be able to walk without help at a later age than their peers, while others can only manage walking with help.

Developmental delay appears to be present in only about half of the documented spEDS cases (26/49). In many instances, psychomotor development is only mildly delayed, possibly because of the confounding effect of minor behavioral abnormalities. Of our three patients, Pt. 1 IQ has been demonstrated to be average, while Pt. 2 and 3 require a support teacher during school only because of a diminished ability to concentrate, and actually have good comprehensive and expressive skills.

In summary, among all of these clinical features, the most relevant in order to suspect spEDS, because of their rarity in other syndromic conditions, are:

- the extreme distal joint hypermobility and soft, hyperextensible skin, particularly of the hands;
- the radiological signs, which are the main indicator for discriminating spEDS-*B4GALT7*, associated with radioulnar synostosis, and spEDS-*B3GALT6*, characterized by kyphoscoliosis (congenital or early onset and progressive) and by the skeletal signs of SEMDJL1 (platyspondyly, short iliac bones, elbow dislocation).

Current EDS classification includes a third type of spEDS, caused by biallelic defects in SLC39A13 (OMIM *608735), which encodes a zinc transporter involved in connective tissue development [57]. (Radiological findings suggest a minor skeletal involvement compared to other spEDS, but overall,) the small number of reported families is inadequate to discuss a differential diagnosis with good confidence [58,59].

The differential diagnosis of spEDS-*B3GALT6* may be placed with kyphoscoliotic EDS (kEDS), primarily owing to early onset kyphoscoliosis, but the two conditions are very different from the clinical point of view, because the latter is not characterized by significantly short stature, and the joint hypermobility with dislocations/subluxations involves mainly shoulders, hips and knees rather than hands and feet.

The differential diagnosis may be placed also with musculocontractural EDS (mcEDS), but the latter is characterized by congenital multiple contractures, characteristically adduction-flexion contractures, and typical craniofacial features, which are distinct from spEDS.

The main differential diagnosis of spEDS-*B4GALT7* can be given with arthrochalasia EDS (aEDS), which is characterized by congenital bilateral hip dislocation and laxity of small joints, but generally aEDS also presents subluxation of the knees and dorso-lumbar kyphosis.

A clinician should consider the differential diagnosis between spEDS-*B4GAL7* and the *B3GAT3*-related linkeropathy, mainly because of prominent eyes, short stature with joint laxity and radioulnar synostosis. However, it should be noted that patients with *B3GAT3* variants often have cardiovascular abnormalities, and that the rarity of the cases described in the literature does not allow definite conclusions [14,15]. Additional clinical descriptions of these disorders are required in order to characterize the linkeropathies both individually and as a disease group.

The radiological feature of spEDS may further warrant consideration for differential diagnosis with several other skeletal dysplasias. The main discriminating features of *B4GALT7*- and *B3GALT6*-related conditions, i.e. respectively radioulnar synostosis and scoliosis with early onset and rapid evolution, can be found in various skeletal dysplasias, but are associated with such a remarkable degree of joint hypermobility and soft, hyperextensible skin almost exclusively in spEDS.

## 5. Conclusions

The most striking aspect of spEDS is the combination of clinical signs affecting both the connective tissue and the skeletal system. The criteria identified in the International Classification of EDS by Malfait et al. (2017) clearly show that there is significant overlap between the clinical features of spEDS-*B4GALT7* and spEDS-*B3GALT6*. This is confirmed and expanded upon by the clinical reports and literature review tables (Table 1 and Table 2) presented here.

Overall, although skin and joints are similarly affected in both conditions, *B3GALT6* mutations lead to a more extensive and severe involvement of the skeletal system, with features often found in SEMDJL1 such as kyphoscoliosis, platyspondyly, short iliac bones and elbow disclocation.

Careful observation of the hands, with their very soft and hyperextensible skin and extreme distal joint laxity, in combination with the specific radiological signs, can immediately evoke the suspicion of either spEDS-*B4GALT7* (radioulnar synostosis) or spEDS-*B3GALT6* (severe progressive kyphoscoliosis and other SEMDJL1-like skeletal features).

The extreme hypermobility of distal joints and the soft, doughy skin on the hands and feet are rarely seen in other EDS types (except, to some degree, in kEDS and aEDS) and are a valuable clue for the diagnosis, which should be supported by skeletal radiographs and by molecular analysis.

Once spEDS is suspected, direct Sanger sequencing is still a viable and cost-effective option for molecular analysis, since *B4GALT7* and *B3GALT6* have short coding regions and only a few exons (6 and 1, respectively). This should be considered especially if the radiological signs restrict the hypothesis to either gene. However, particularly when attempting a molecular analysis early on, when some of the distinctive clinical signs have not yet evolved, an NGS panel specific for EDS would be advisable, since it can help confirm the differential diagnosis.

Being able to distinguish between spEDS-*B3GALT6* and spEDS-*B4GALT7* is important for clinicians, because some patients in the *B3GALT6*-related group may develop life-threatening complications such as aortic dilatation, aneurysms and cervical spine instability.

In conclusion, accurate diagnosis will help in excluding other causes of peripheral hypotonia, such as neuromuscular disorders, and allow for appropriate physiotherapeutic interventions. 

## Figures and Tables

**Table 1:** Patients with *B4GALT7* variants: Review of the literature.

		Hernandez et al. [1979, 1981, 1986] [21,22,23]	Kresse et al. [1987][24]	Faiyaz-Ul-Haque et al. [2004][29]	Guo et al. [2013][30]	Cartault et al. [2015][31]	Arunrut et al. [2016][33]	Salter et al. [2016][32]	Ritelli et al. [2017][51]	Sandler-Wilson et al. [2019][52]	This study, Patient 1	Total (patients with mutation)
**Genetics**	Diagnosis (Various denominations)	A distinct variant of the EDS	EDS, progeroid type 1	EDS, progeroid type 1	EDS, progeroid type 1	Larsen of Reunion Island syndrome	*B4GALT7-*linkeropathy phenotype	Phenotypic Spectrum of *B4GALT7*	spEDS- *B4GALT7*	spEDS- *B4GALT7*	spEDS- *B4GALT7*	spEDS- *B4GALT7*
	*B4GALT7* variants	?	c.[557C>A ];[617T>A]	c.[808C>T];[808C>T]	c.[122T>C];[808C>T]	c.[808C>T];[808C>T]	c.[970T>A];[970T>A]	c.[277dupC];[641G>A] c.[421C>T];[808C>T]	c.[829G>T];[829G>T]	c.[421C>T];[808C>T]	c.[277_278insC];[628C>T]	26/33 Homozygous 7/33 Compound heterozygous
	Variants on protein	?	p.[(A186D)];[(L206P)]	p.[(R270C)];[(R270C)]	p.[(L41P)];[(R270C)]	p.[(R270C)];[(R270C)]	p.[(C324S)];[(C324S)]	p.[(H93Pfs*73)];[(C214Y)] p.[(R141W)];[(R270C)]	p.[(E277*)];[(E277*)]	p.[(R141W)];[(R270C)]	p.[(H93Pfs*73)];[(H210Y)]	
	Gender	5M	M	1F 1M	M	11M 11F	F	1M 1F	F	1M 1F	M	17M 16F
	Age	8y, 15y, 15y, 16y, 18y	4y 9m	2y, 33y	10y	4y, 46y	5y	3y 6m, 13y	30y	4y, 10y	7y 8m	2y → 46y
**Main features**	Short stature^a^	4/5	+	2/2	+	22/22	+	2/2	+	2/2	+	33/33
	Radiolunar synostosis^c^	n.a.	+	2/2	+	10/21	-	2/2	-	2/2	+	19/32
	Bowing of limbs^a^	n.a.	+	2/2	+	21/21	-	2/2	-	2/2	+	30/32
	Joint hypermobility especially of the hands^c^	5/5	+	2/2	+	22/22	+	2/2	+	2/2	+	33/33
	Skin hyperextensibility, soft, doughy skin^b^	5/5	+	2/2	+	21/22	+	2/2	+	2/2	+	32/33
**Facial dysmorphisms^c^**	Progeroid facial appearance	5/5	mild	0/2	-	0/22	-	0/2	-	0/2	-	1/33
	Short face	-	+	2/2	-	22/22	+	2/2	-	2/2	+	31/33
	Midface hypoplasia	-	-	2/2	-		+	2/2	-	2/2	+	30/33
	Narrow mouth	-	+	2/2	-		+	1/2	+	2/2	+	31/33
	Proptosis	-	+	2/2	-		+	2/2	+	2/2	+	32/33
	Cleft palate	-	Bifid uvula	0/2	-	1/22	-	1/2	-	1/2	-	4/33
	Loose skin	-	+	2/2	+	n.a.	n.a.	2/2	+	n.a.	+	8/8
**Other clinical features**	Delayed wound healing	5/5	+	1/2	+	n.a.	+	2/2	+	n.a	-	7/8
	Cardiovascular abnormalities	1/5 Aortic/Pulmonic Stenosis	n.a.	n.a.	-	n.a.	-	n.a.	-	n.a.	+ :ASD	1/4
	Delayed motor development^b^	5/5	+	2/2	-	n.a.	+	2/2	+	2/2	+	10/11
	Delayed cognitive development^b^	5/5	n.a.	n.a.	Mild learning disabilities	12/22 (learning disabilities)	n.a.	1/2 Severe 1/2 n.a	-	2/2	-	16/29
	Muscle hypotonia^a^	n.a.	+	2/2	mild	n.a.	+	2/2	+	2/2	+	11/11
	Ophthalmological abnormalities^c^	n.a.	-	1/2 Mild esotropia and mild hypermetropia	Severe hyperopia, congenital ptosis, intermittent exotropia	5/21 glaucoma 1/21 megalocornea	Nystagmus Iris and optic nerve colobomas Posterior subcapsular cataracts High hyperopia Right-sided ptosis	Severe hypermetropia Small optic nerves Hypermetropia Strabismus	-	2/2 Blue sclerae 1/2 Severe hyperopia	Myopia	13/32
	Osteopenia^b^	n.a.	+	2/2	-	n.a.	n.a.	2/2	+	1/2	+	8/10
	Pes planus^b^	5/5	+	½	+	n.a.	+	1/2 1/2 n.a.	+	2/2	+	9/11
	Bilateral elbow contractures or limited elbow movement^c^	n.a.	+	2/2	+	n.a.	n.a.	2/2	-	1/2	+	8/10
	Sensorineural hearing loss	n.a.	-	n.a.	n.a.	n.a.	n.a.	1/2 Conductive hearing loss	+	n.a.	-	2/7
	Other less frequent features	Cryptorchidism 4/5 Inguinal hernia 1/5 Hypogonadism 1/5 Varicose veins 5/5 Multiple nevi 5/5 Dental anomalies 5/5	Dental anomalies: defective and greyish enamel Clavicular exostoses	Yellow discoloration of teeth with defective enamel Mild eventration of the right hemidiaphragm Bilateral equinovarus deformity	Unilateral ptosis	Pectus carinatum 5/22 Bifid thumb 2/22 Scoliosis/ Kyphosis 6/22	Pectus carinatum Scoliosis Broad fingertips Subluxation of the distal interphalangeal joints Absence of the pineal gland Prominent scalp veins	Irregular and fragile dentition Scoliosis Bilateral patellar dislocation	Bilateral hallux valgus Lymphedema Scoliosis Temporomandibular joint dislocation	1/2 Chest wall deformity 2/2 Coronal cleft vertebrae 2/2 Sagittal craniosynostosis 1/2 Vesicoureteral reflux	Hip dysplasia Ulnar deviation of fingers	

EDS: Ehlers–Danlos syndrome; n.a. not available; ASD: atrial septal defect; ^a^Spondylodysplastic EDS (spEDS) major criteria [46]; ^b^spEDS minor criteria [46]; ^c^spEDS specific minor criteria for *B4GALT7* variants [46]. The 5 patients from Hernandez et al. (column 1) had clinical data compatible with spEDS-*B4GALT7*, but were not considered in the clinical signs totals because it was unclear whether the clinical diagnosis was confirmed by molecular testing or not [21,22,23].

**Table 2:** Patients with *B3GALT6* variants: Review of the literature.

		Malfait et al. 2013[44]	Nakajima et al. 2013[45]	Sellars et al. 2014[53]	Ritelli et al., 2015[54]	Alazami et al., 2016[18]	Trejo et al.2017[55]	Ben-Mahmoud et al. 2018[56]	Van Damme et al. 2018[48]	This study, Patient 2	This study, Patient 3	Total (patients with mutation)
**Genetics**	Diagnosis (Various denominations)	EDS-like connective tissue disorder SEMDJL1	B3GALT6 spectrum SEMDJL1 EDS-progeroid form	EDS progeroid type2 SEMDJL1	EDS-like syndrome EDS progeroid type2 SEMDJL1	B3GALT6- phenotype	SEMDJL1	spEDS- B3GALT6 SEMDJL1	spEDS- B3GALT6	spEDS- B3GALT6	spEDS- B3GALT6	spEDS- B3GALT6
	*B3GALT6* variants	c.[619G>C];[619G>C] c.[323_344del];[619G>C] c.[649G>A];[649G>A]	9 Compound heterozygous, 1 Heterozygous; see Ref. (Table 3)	?	c.[227delT];[766C>T]	c.[556T>C];[556T>C] c.[536_541dup];[536_541dup]	c.[511C>T];[901_921dup]	c.[618C>G];[618C>G]	8 Compound heterozygous, 1 Homozygous; see Ref (Table 3)	c.[353delA];[925T>A]	c.[308C>T;353delA];[987_989delCTG]	12/45 homozygous 32/45 compound heterozygous 1/45 heterozygous
	Variants on protein	p.[(D207H)];[(D207H)] p.[(A108Gfs*163)];[(D207H)] p.[(G217S)];[(G217S)]	See Figure 7	p.[(D159Y)];[(E265D)]	p.[(I76Tfs*202)];[(R256W)]	p.[(F186L)];[(F186L)] p.[(R179_R180dup)];[(R179_R180dup)]	p.[(R171C)];[(K301_R307dup)]	p.[(C206W)];[(C206W)]	See Figure 7	p.[(D118Afs*160)];[(S309T)]	p.[(A103V);(D118Afs*160)];[(*330Aext*73)]	
	Gender	2M 3F	6M 6F	M	2F	1M 4F	3F	1M 2F	7M 5F	M	M	20M 25F
	Age	1y8m →27y	1m →34y	6m	21y, 25y	6w → 6y	12y, 15y, 15y	4d →2m	8m →37y	12y 7m	13y 3m	4d →37y
**Main features**	Short stature ^a^	2/3	12/12	+	2/2	3/3	3/3	n.a.	10/10	+	+	35/36
	Kyphoscoliosis(congenital or early onset, progressive) ^c^	3/4	12/12	-	2/2	4/5	3/3	n.a.	10/10	+	+	36/39
	Bowing of limbs ^a^	2/4	n.a.	+	n.a.	n.a.	3/3	n.a.	10/10	-	-	16/20
	Joint hypermobility especially of the hands ^c^	4/4	7/10	n.a.	2/2	5/5	3/3	2/2	10/10	+	+	33/36
	Skin hyperextensibility, soft, doughy skin ^b^	4/4	6/10	n.a.	2/2	4/5	3/3	2/2	9/10	+	+	33/38
	Skeletal changes SEMDJL1 ^d^	3/4	12/12	n.a.	2/2	2/2	0/1	3/3	10/10	+	+	34/36
**Facial dysmorphisms^c^**	Prominent forehead	4/4	9/10	+	2/2	4/5	0/3	1/1	8/10	+	+	31/38
	Sparse hair	2/4	3/10	-	0/2	n.a	2/3	0/1	3/3	+	+	12/26
	Midface hypoplasia	2/4	n.a.	+	1/2	4/5	2/3	1/1	8/10	+	+	21/28
	Blue sclerae	3/4	7/10	n.a.	2/2	4/5	1/3	n.a.	6/10	+	+	25/36
	Proptosis	2/4	7/10	+	0/2	n.a.	3/3	n.a.	7/10	-	-	20/32
	Cleft palate	0/4	1/10	-	0/2	n.a.	0/3	-	-	-	-	2/25
**Other clinical features**	Joint hand contractures ^c^	2/3	3/12	+	2/2	1/5	2/2	3/3	10/10	-	-	24/40
	Cardiovascular anomalies	n.a.	Mitral regurgitation 1/?	n.a.	2 Mitral valve prolapse	1 Aortic valve stenosis 1 Mitral valve prolapse/?	n.a.	n.a.	Aortic root aneurysm 3/8 Cardiac valve anomalies 2/8	Aortic root aneurysm Mitral valve prolapse	Mitral valve prolapse	12/?
	Delayed motor development ^b^	4/4	2/?	+	n.a.	4/5	2/2	n.a.	5/9	+	+	17/24
	Delayed cognitive development ^b^	2/2	n.a.	n.a.	0/2	4/5	n.a.	n.a.	3/8	-	-	10/20
	Muscle hypotonia ^a^	4/4	5/12	+	1/1	2/?	3/3 (1 mild)	1/1	5/9	+	+	25/37
	Ophthalmological anomalies	Myopia 2/4 Retinal detachment1/4	n.a.	Corneal opacity Sclerocornea	0/2	n.a.	n.a	Corneal opacity 3/3	Glaucoma and optic nerve atrophy 1/10 Microcornea 1/10	-	-	7/19
	Osteopenia ^b^	4/4	n.a.	+	2/2	3/3	0/3	2/2	8/8	+	+	22/26
	Pes planus ^b^	2/2	n.a.	n.a.	2/2	n.a.	n.a	0/2	n.a.	+	+	6/8
	Talipes equinovarus ^c^	3/4	4/12	+	n.a	3/5	n.a	2/2	10/10	+	+	23/37
	Peculiar fingers ^c^	3/4	7/11	+	2/2	n.a.	n.a	2/2	n.a.	+	+	17/22
	Anomalies of dentition, discoloration of teeth ^c^	3/4	n.a.	n.a.	2/2	n.a.	n.a	n.a.	8/9	+	+	13/17
	Less frequent features	Excessive wrinkling of palmar skin (hands and feet) 2/4 Pectus deformity 3/4	Elbow dislocation 9/10 Limited elbow movement 9/11 Carpal synostosis 1/10 Short metacarpals 6/10 Hip dislocation 5/12 Epiphyseal dysplasia of femoral head 4/12	Radioulnar synostosis Early death	2/2 Genu valgus 2/2 Hallux valgus	4/5 Multiple fractures Bilateral disclocated radial head 1/5 Pectus carinatum	3/3 Bilateral radioulnar dislocation 3/3 Hip dysplasia 2/3 Hearing loss 1/3 Pectus carinatum 1/3 Ulnar deviation of the fingers	Contractures of the large joints 2/2 Radioulnar synostosis 2/2 Oligodactyly of the right 3rd finger 1/2 Spontaneous fractures 2/2 Early death 3/3	Sensorineural and conductive hearing loss 1/10 Cervical spine instability 3/7 Laryngeal cleft 1/10 Tracheomalacia 2/10 Spontaneous repeated pneumothoraces 1/10 Chronic respiratory insufficiency 2/10 Pectus carinatum 1/10 excavatum 1/10 Wilms tumor 1/10 Joint dislocations 10/10 Hip dysplasia 4/6 Fractures 8/9 Hallux valgus 3/10	Prominent superficial veins Limited elbow extension Ptosic kidney, Bilateral caliceal and ureteral dilatation Fractures Right hip dysplasia Recurrent luxation of the toes, Hypoplastic nails Hallux valgus	Prominent superficial veins Barrett’s oesophagus Limited elbow extension Bilateral cryptorchidism Hypoplastic nails	

EDS: Ehlers Danlos syndrome; n.a. not available; ASD: atrial septal defect; ^a^Spondylodysplastic EDS (spEDS) major criteria [46]; ^b^spEDS minor criteria [46]; ^c^spEDS specific minor criteria for *B3GALT6* variants[46]; ^d^ Platyspondyly, short ilia, elbow malalignment. Cases from 10 additional families reported by Vorster et al. in 2015 [42] were not listed here due to difficult data interpretation; they include an additional variant, c.235A>C, p.(T79A).
